# Proteomic analysis of blastema formation in regenerating axolotl limbs

**DOI:** 10.1186/1741-7007-7-83

**Published:** 2009-11-30

**Authors:** Nandini Rao, Deepali Jhamb, Derek J Milner, Bingbing Li, Fengyu Song, Mu Wang, S Randal Voss, Mathew Palakal, Michael W King, Behnaz Saranjami, Holly LD Nye, Jo Ann Cameron, David L Stocum

**Affiliations:** 1 Department of Biology and Center for Regenerative Biology and Medicine, Indiana University-Purdue University Indianapolis, Indianapolis, IN, USA; 2 School of Informatics and Center for Regenerative Biology and Medicine, Indiana University-Purdue University Indianapolis, Indianapolis, IN, USA; 3 Department of Cell and Developmental Biology, and Regeneration Biology and Tissue Engineering Theme, Institute for Genomic Biology, University of Illinois-Urbana Champaign, Urbana, IL, USA; 4 Department of Oral Biology, School of Dentistry and Center for Regenerative Biology and Medicine, Indiana University-Purdue University Indianapolis, Indianapolis, IN, USA; 5 Department of Biochemistry, School of Medicine and Center for Regenerative Biology and Medicine, Indiana University-Purdue University Indianapolis, Indianapolis, IN, USA; 6 Department of Biology and Spinal Cord and Brain Injury Center, University of Kentucky at Lexington, Lexington, KY, USA

## Abstract

**Background:**

Following amputation, urodele salamander limbs reprogram somatic cells to form a blastema that self-organizes into the missing limb parts to restore the structure and function of the limb. To help understand the molecular basis of blastema formation, we used quantitative label-free liquid chromatography-mass spectrometry/mass spectrometry (LC-MS/MS)-based methods to analyze changes in the proteome that occurred 1, 4 and 7 days post amputation (dpa) through the mid-tibia/fibula of axolotl hind limbs.

**Results:**

We identified 309 unique proteins with significant fold change relative to controls (0 dpa), representing 10 biological process categories: (1) signaling, (2) Ca^2+ ^binding and translocation, (3) transcription, (4) translation, (5) cytoskeleton, (6) extracellular matrix (ECM), (7) metabolism, (8) cell protection, (9) degradation, and (10) cell cycle. In all, 43 proteins exhibited exceptionally high fold changes. Of these, the ecotropic viral integrative factor 5 (EVI5), a cell cycle-related oncoprotein that prevents cells from entering the mitotic phase of the cell cycle prematurely, was of special interest because its fold change was exceptionally high throughout blastema formation.

**Conclusion:**

Our data were consistent with previous studies indicating the importance of inositol triphosphate and Ca^2+ ^signaling in initiating the ECM and cytoskeletal remodeling characteristic of histolysis and cell dedifferentiation. In addition, the data suggested that blastema formation requires several mechanisms to avoid apoptosis, including reduced metabolism, differential regulation of proapoptotic and antiapoptotic proteins, and initiation of an unfolded protein response (UPR). Since there is virtually no mitosis during blastema formation, we propose that high levels of EVI5 function to arrest dedifferentiated cells somewhere in the G_1_/S/G_2 _phases of the cell cycle until they have accumulated under the wound epidermis and enter mitosis in response to neural and epidermal factors. Our findings indicate the general value of quantitative proteomic analysis in understanding the regeneration of complex structures.

## Background

With the exception of cervid antlers [1,2], terminal phalanges of humans and rodents [3-5], and ear tissue of certain strains of mice and rabbits, [6,7], mammalian appendages do not regenerate after amputation. By contrast, urodele salamanders have the unique natural ability to regenerate appendages from any level of amputation by the formation of a blastema that subsequently self-organizes into the amputated limb parts [8-10]. After amputation, proteolysis of extracellular matrix (ECM) liberates muscle, skeletal, connective tissue, and peripheral nerve Schwann cells from their tissue organization [11]. The liberated cells dedifferentiate and migrate under the wound epidermis to form an avascular accumulation (also called early bud) blastema [12-14]. In addition, satellite cells contribute to muscle formation in the blastema [15,16], and it would not be surprising if mesenchymal stem cells of the periosteum and endosteum contributed to the blastema as well. Blastema cells morphologically resemble mesenchymal stem-like cells, although their surface antigens and other biomarkers are incompletely characterized. Once formed, the accumulation blastema is enlarged to the medium bud stage and beyond by a marked increase in mitosis [17-23]. Sustained mitosis of blastema cells, but not dedifferentiation, is dependent on factors from the wound epidermis [21] and regenerating nerves [24]. Histological [17,18], cell marking [25,26] and genetic marking [27] studies indicate that blastema cells derived from each tissue redifferentiate into the same tissue, although some cells derived from the dermis differentiate into cartilage as well.

Analysis of the molecular mechanisms of blastema formation in the urodele limb is useful for understanding how we might achieve the goal of mammalian regeneration *in situ *by chemical induction [28]. The traditional approach to molecular research on amphibian limb regeneration has been to characterize the expression patterns and functional roles of single genes expressed during embryonic limb development. A large number of genes have been studied in this way, particularly genes involved in pattern formation [10,29,30]. Less biased and more global analyses have recently been conducted using subtractive hybridization and microarrays to compare transcriptional profiles of regenerating versus intact limb tissues, or to compare blastemas of regeneration-competent versus regeneration-deficient limbs [31-35].

A number of studies have been carried out on protein synthesis and separation in regenerating urodele limbs. Autoradiographic studies of C^14 ^methionine, S^35 ^thioamino acids or C^14 ^leucine incorporation revealed intense protein synthesis throughout regeneration [36-41]. Several protein separation analyses have been carried out using one-dimensional or two-dimensional gel electrophoresis [42-45]. These resolved up to 800 individual proteins [44] and revealed differences in protein composition at succeeding stages of regeneration in normal [43,44] and denervated limbs [42], although few proteins were identified.

Protein separation and identification technology has evolved rapidly in the past 5 years with the introduction of label-free liquid chromatography/mass spectrometry methods that can more accurately identify and quantify peptide species. Also, with the development of expressed sequence tag (EST) databases [46,47], it is possible to annotate short peptide sequences to protein models. Here, we report the application of this technology to analyze the formation of the accumulation blastema in regenerating axolotl hind limbs. Our results confirm a number of earlier studies on signaling, cytoskeletal and ECM changes, and metabolism. They also suggest that the amputated urodele limb uses a combination of mechanisms to regulate apoptosis during blastema formation that might be essential for dedifferentiation. Lastly, we have identified a highly upregulated centrosomal cell cycle-related oncoprotein, ecotropic viral integrative factor 5 (EVI5), that may play a key role in preventing dedifferentiated cells from entering mitosis until an accumulation blastema has formed.

## Results

### Histology

**Figure 1:**
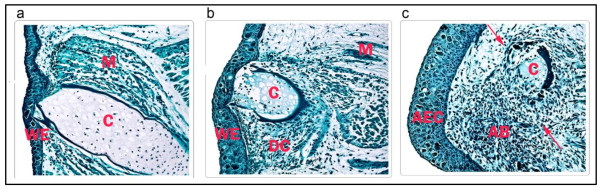
**Histology of axolotl hindlimbs**. Longitudinal sections of axolotl hindlimbs regenerating from the mid-tibia/fibula, stained with Weigert's iron hematoxylin and light green SF: **(a) **Sections at 1 day post amputation (dpa). The amputation surface is covered with several layers of wound epidermal (WE) cells, including gland cells. The basal layer of the wound epidermis is in direct contact with underlying tissues. Some cell debris, red blood cells and lymphocytes are present under the wound epithelium. C = cartilage, M = muscle. **(b) **Sections at 4 dpa. The cartilage (C), muscle (M), and dermal tissue organization is breaking down, releasing cells that dedifferentiate (DC) and migrate toward the wound epithelium (WE). **(c) **Sections at 7 dpa. Blastema cells have accumulated under a thickened apical epidermal cap (AEC) to form an accumulation blastema (AB). C = cartilage. The arrows indicate the junction between the accumulation blastema and tissues still undergoing dedifferentiation. Magnification = 10 ×.

Figure 1 shows the histological appearance of regenerating limbs in longitudinal section at 1, 4 and 7 dpa. At 1 dpa, the wound epidermis, including gland cells, has migrated to cover the wound. Clotted plasma, muscle fragments, cellular debris and lymphocytes are present under the wound epidermis, which is 3 to 4 cells thick. At 4 dpa, histolysis is liberating cells from their tissue organization and blastema cells have begun to accumulate under the wound epidermis. Osteoclasts can be seen eroding the matrix of the periosteal bone shell that surrounds the cartilage. The basement membrane under the wound epidermis is absent and the wound epidermis is in direct contact with the underlying tissues. By 7 dpa, further histolysis and distal migration of dedifferentiated cells has produced an avascular accumulation blastema with a cell density distinctly higher than that of the more proximal tissue. Examination of serial sections revealed few mitotic figures from 1 to 7 dpa. Some pyknotic nuclei were observed at 1 dpa, but not at 4 and 7 dpa, suggesting a minimum of cell death during the latter time frame.

### Proteomic analysis

A total of 1,624 peptides were separated in the samples. In all, 138 from priority 1 and 285 peptides from priority 2 were statistically significant (Additional file 1). Of these 423 statistically significant peptides, 114 peptides were not analyzed further for the reasons outlined in Methods. A total of 309 proteins (Additional file 2) were analyzed for their role in biological processes. A comparison of non-redundant peptide sequences (N = 405) with the axolotl EST database identified 149 perfect-match peptides (36.8%) that were 100% identical to a translated EST contig from either *Ambystoma mexicanum *or the closely related *Ambystoma tigrinum*. These proteins are shown in bold in Additional file 1.

**Figure 2:**
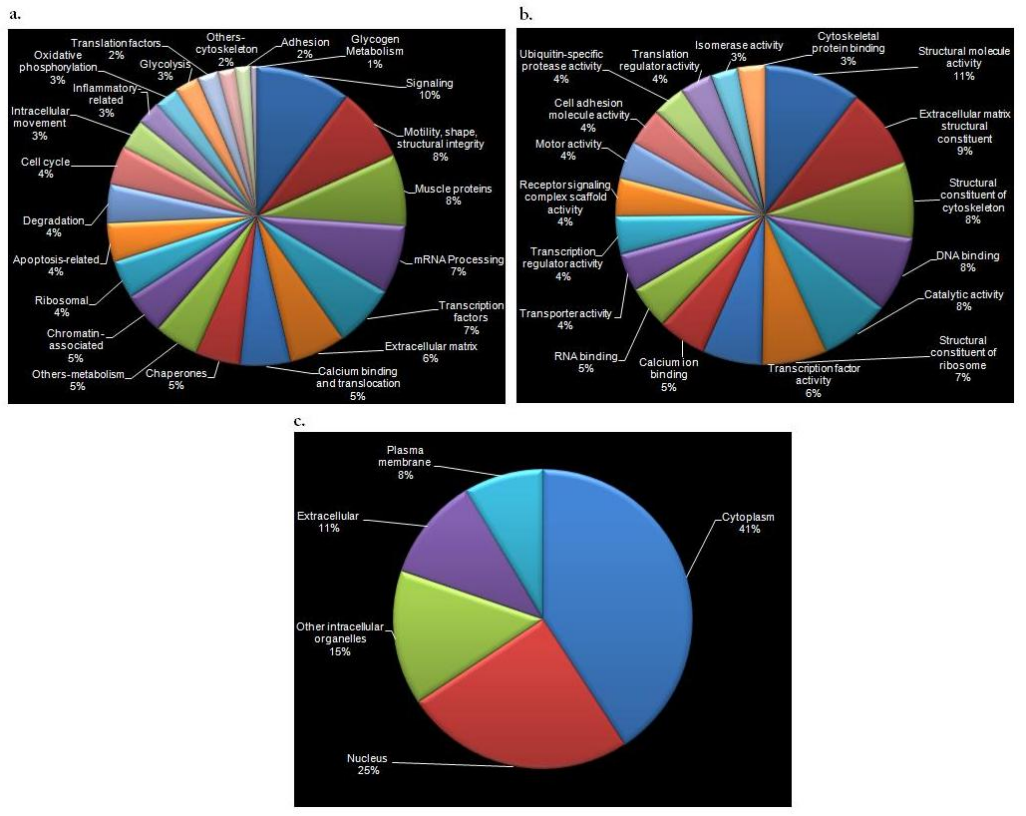
**Functional and cellular categorization of proteins**. Pie charts showing categories of 309 proteins according to **(a) **biological process, **(b) **molecular function, and **(c) **cellular location. Only the categories with at least five proteins have been included in the molecular function pie chart. Since a large number of categories were obtained from the Human Protein Reference Database (HPRD) for cellular localizations, they were classified into five major categories: cytoplasm (actin cytoskeleton, cytosol, and clathrin-coated vesicle), nucleus (centrosome, chromosome, and nucleolus), other intracellular organelles (ribosome, sarcoplasmic reticulum, sarcoplasm, mitochondrial matrix, mitochondrial membrane, mitochondrion, endoplasmic reticulum, golgi apparatus, lysosome and peroxisome), plasma membrane (integral to membrane) and extracellular (cell junction, extracellular matrix).

**Figure 3:**
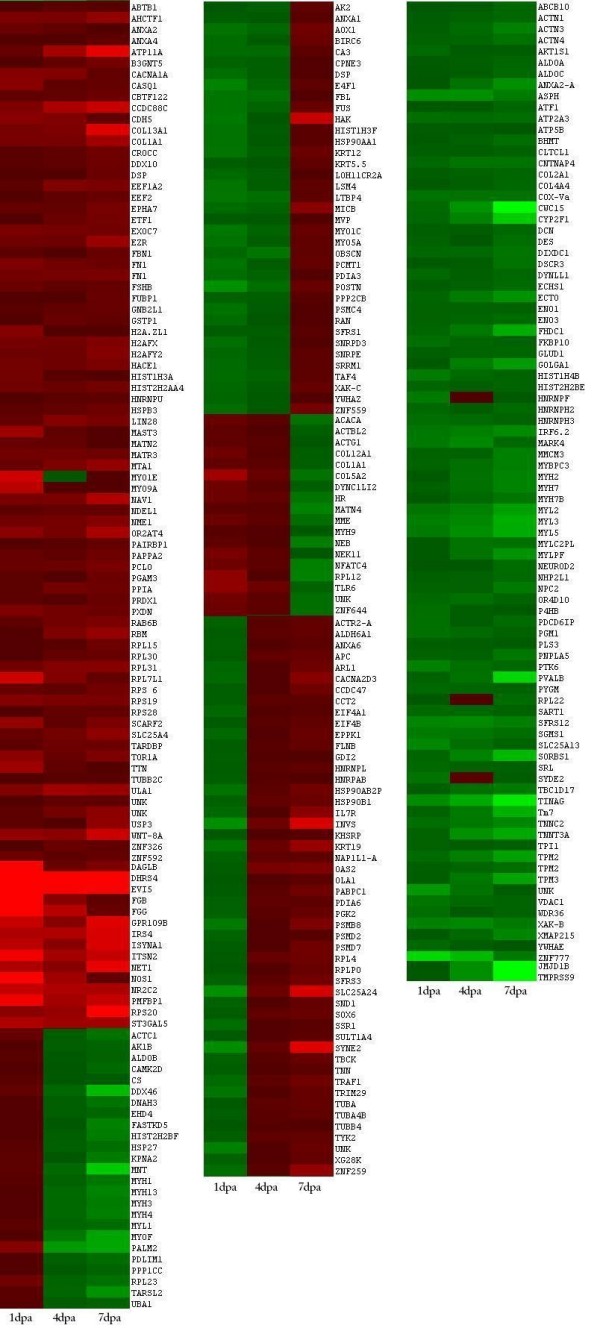
**Global Expression intensity map**. HeatMap showing upregulation (red) and downregulation (green) of priority 1 and 2 proteins identified as having significant fold changes relative to control. Numbers at bottom of each column indicate days post amputation (dpa). Left column: proteins upregulated on all dpa, or 1 dpa. Middle column: proteins downregulated on 1 and 4 dpa, and upregulated at 7 dpa; proteins upregulated at 1 and 4 dpa and downregulated at 7 dpa; and proteins downregulated at 1 dpa and upregulated at 4 and 7 dpa. Right column: proteins downregulated on all dpa or two of three dpa. Color intensity reflects fold change.

Figure 2 stratifies the proteins according to biological process, molecular function and cellular location. Figure 3 is a global intensity map of fold changes at 1, 4 and 7 dpa. The peptides were grouped into 10 biological process categories (see Additional file 2): (1) signaling, (2) Ca^2+ ^binding and translocation, (3) transcription, (4) translation, (5) cytoskeleton, (6) ECM, (7) metabolism, (8) cell protection, (9) degradation, and (10) cell cycle. Table 1 summarizes the ratios of the numbers of proteins upregulated (U) to downregulated (D) relative to controls (U/D ratios) at 1, 4, and 7 dpa for each category of biological process. Table 2 lists those proteins with positive or negative fold changes greater than two with respect to controls, and which may thus have special biological significance. Below, we describe the results for each of the 10 biological categories in order, with those proteins upregulated or downregulated by a factor of 2.0 or more at any time point shown in bold type.

**Table 1:** Upregulation/downregulation ratios

Biological process	1 day	4 days	7 days
Signaling associated (34)	0.72	1.72	2.75

Ca^2+ ^binding and translocation (17)	0.36	0.63	1.29

Transcription and translation (78)	0.79	1.52	2.04

Transcription (58)	0.66	1.08	1.65

Chromatin associated (14)	1.60	1.60	1.40

Transcription factors (21)	0.9	1.57	2.00

mRNA processing (23)	0.24	0.62	1.50

Translation (20)	1.50	7.00	4.00

Ribosomal proteins (13)	2.33	9.00	3.33

Translation factors (7)	0.67	5.00	6.00

Cytoskeleton (68)	0.42	0.31	0.42

Muscle (23)	0.18	0.00	0.04

Non-muscle proteins (45)	0.58	0.60	0.85

Motility, shape, structural integrity (25)	0.28	1.00	1.44

Intracellular Movement (10)	1.00	0.11	0.33

Adhesion (5)	1.50	1.50	1.50

Other (5)	3.00	0.66	0.66

Extracellular matrix (19)	2.00	1.83	1.29

Metabolism (33)	0.35	0.29	0.45

Oxidative phosphorylation (8)	0.33	0.17	0.14

Glycolysis (8)	0.33	0.14	0.33

Glycogen (2)	0.00	0.00	0.00

Other (15)	0.44	0.75	1.00

Cell protection (35)	0.47	1.00	2.30

Inflammation related (7)	1.00	2.50	2.50

Apoptosis related (13)	0.25	0.71	1.40

Chaperones (15)	0.50	0.88	3.70

Degradation (11)	0.86	1.00	2.00

Cell cycle (14)	1.00	0.86	1.20

Ratio of proteins upregulated and downregulated for biological process categories at 1, 4, and 7 days post amputation (dpa). Numbers in parentheses represent numbers of proteins in each category.

**Table 2:** Highly regulated proteins

Biological process	Protein	1 day	4 days	7 days
Signaling associated	CCDC88C	1.52	2.03	2.37
	
	GPR109B	2.38	1.61	2.65
	
	INVS	-1.71	1.32	2.57
	
	IRS4	2.06	2.09	2.56
	
	ISYNA1	2.20	1.64	2.59
	
	ITSN2	2.86	1.96	2.32
	
	NET1	2.06	1.61	2.76
	
	NOS1	4.93*	1.91	1.16
	
	OR2AT4	1.67	138	2.01
	
	WNT8A	1.74	1.65	2.43

Ca^2+ ^binding and translocation	ATP11A	1.21	196	2.72
	
	HAK	-1.43	-1.06	2.37
	
	PVALB	-1.16	-1.35	-2.56
	
	SLC25A24	-1.71	1.32	2.57

Transcription and translation	CWC15	-1.26	-1.74	-3.33*
	
	DDX46	1.18	-1.23	-2.22
	
	JMJD1B	-1.05	-1.67	-6.82
	
	MNT	1.11	-1.26	-2.39
	
	NR2C2	2.32	2.06	2.05
	
	RPL7L1	2.47	1.54	1.17
	
	RPS20	1.63	1.80	3.61*
	
	ZNF777	-2.58	-2.23	-1.43

Cytoskeleton	FHDC1	-1.22	-1.45	-2.06
	
	MYL3	-1.49	-1.62	-2.06
	
	MYL5	-1.45	-1.69	-2.04
	
	MYO1e	2.44	-1.05	NC
	
	MYO9A	2.21	NC	NC
	
	NAV1	1.42	1.45	2.11
	
	PMFBP1	2.86	1.96	2.32
	
	SORBS1	-1.22	-1.57	-2.20
	
	ST3GAL5	2.07	1.87	1.96
	
	SYNE2	-1.68	1.20	2.67
	
	TM7	-1.17	-1.45	-2.10
	
	TNNT3A	-1.17	-1.71	-2.01

Extracellular matrix	COL13A1	1.41	1.49	2.66
	
	FGB	3.39*	1.63	1.14
	
	FGG	4.64*	2.17	1.14
	
	TINAG	-1.68	-2.01	-2.77

Metabolism	DAGLB	3.88*	1.26	1.49
	
	DHRS4	4.45*	3.93*	4.21*

Cell protection	CYP2F1	-1.24	-1.55	-2.42

Degradation	TMPRSS9	-1.07	-1.70	-6.95*

Cell cycle	EVI5	4.00*	3.29*	3.85*

Priority 1 and 2 proteins upregulated or downregulated by a value of 2.00 or more on one or more days post amputation (dpa). Asterisks indicate exceptionally high values (over 3.00) on one or more dpa.

### Signaling

A key intracellular signaling pathway is the inositol triphosphate/diacylglycerol (IP_3_/DAG) pathway. IP_3 _and DAG are cleavage products of phosphatidylinositol-4, 5-bisphosphate (PIP2). A precursor to PIP2 is myoinositol (inositol). Inositol-3-phosphate synthase 1 (ISYNA1) is a key enzyme in the synthesis of inositol from glucose-6-phosphate, and it was upregulated on all dpa. Two regulators of Rho-type guanosine triphosphatases (GTPases) were detected. SYDE2, a GTPase activator, was upregulated at 4 dpa, but downregulated at 1 and 7 dpa, while NET1, a guanine nucleotide exchange factor, was upregulated on all dpa.

Several proteins involved in endocytotic trafficking were identified. CLTCL1, the major protein of the coat of coated pits and vesicles, was downregulated on all dpa. By contrast, ITSN2, which may regulate the formation of clathrin-coated vesicles, was upregulated on all dpa. Several Rab GTPases and associated factors exhibited differential regulation. The Rab family is involved in vesicular trafficking and signaling. RAB6B was upregulated on all dpa and ARL1 and XG28K were downregulated at 1 dpa and upregulated at 7 dpa. A Rab GTPase activator, TBCK, was upregulated at 4 and 7 dpa, while another, TBC1D17, was downregulated on all dpa. GDI2, which regulates the exchange reaction of most Rab proteins by inhibiting the dissociation of guanidine dihydrogen phosphate (GDP) from them, was downregulated at 1 dpa, then returned to control value at 4 and 7 dpa.

Other signaling-related proteins that were upregulated on all three or two of three dpa were: (1) EZR, a peripheral membrane protein that may act to organize transmembrane receptors and binds to signal transduction molecules such as phosphoinositol 3 (PI3) kinase, (2) the receptor for nicotinic acid GPR109B, (3) IRS4, which interfaces between many growth factors and intracellular signaling molecules, (4) TYK2, which phosphorylates receptors of the Janus kinase (JAK)/signal transducer and activator of transcription (STAT) pathway to transduce cytokine signals, (5) guanine nucleotide binding protein β polypeptide 2-like 1 (GNB2L1), which anchors protein kinase C to the cytoskeleton, (6) EPHA7, the receptor for the A1 to 5 members of the ephrin A family of ligands, and (7) neuronal nitric oxide synthase (NOS1), the enzyme that synthesizes nitric oxide (NO), a gas with a wide variety of signaling functions. Of all the proteins detected, NOS1 exhibited the highest upregulation at 1 dpa (4.93), after which the level of upregulation declined below 2.0 at 4 and 7 dpa. PPP2CB, the catalytic subunit for phosphatase 2A, a major serine/threonine phosphatase implicated in the negative control of cell growth and division, was downregulated at 1 and 4 dpa, and upregulated at 7 dpa. YWHAZ, an adaptor protein that mediates signal transduction by binding to phosphoserine-containing proteins, was also downregulated at 1 and 4 dpa, with no change at 7 dpa. Another adaptor protein, YWHAE, as well as IRF6.2 and tyrosine-protein kinase 6 (PTK6), were downregulated at all dpa. PTK6 is a cytoplasmic protein kinase that may function as an intracellular signal transducer in epithelia.

Five proteins associated with Wnt signaling were detected. Wnt8a, a ligand for the canonical Wnt pathway, was upregulated on all three dpa. Adenomatous polyposis coli (APC), part of the complex that destabilizes β-catenin in the canonical pathway was upregulated at 4 and 7 dpa. CCDC88C, a Disheveled-binding protein that negatively regulates the canonical pathway, was upregulated on all dpa, while DIXDC1, a positive effector of the canonical pathway, was downregulated on all dpa. Inversin (INVS), which acts to switch Wnt signaling from the canonical to the non-canonical pathway by targeting the Disheveled protein for degradation by the ubiquitin proteasome, was upregulated at 4 and 7 dpa.

Two olfactory receptors were detected, with opposite fold change. OR2AT4 was upregulated on all dpa, whereas OR4D10 was downregulated on all dpa. Follicle stimulating hormone receptor (FSHB) was also upregulated on all days. The potential functions of these proteins in limb regeneration are unknown. The latent transforming growth factor (TGF)β-binding protein was upregulated at 7 dpa, and ectodermin (ECTO), a SMAD4 ubiquitin ligase that attenuates the TGFβ response was downregulated on all dpa.

### Ca^2+ ^binding and translocation proteins

The cell maintains cytosolic calcium homeostasis by channels that translocate Ca^2+ ^between the cytosol and the endoplasmic reticulum (ER) or sarcoplasmic reticulum (SR), and between the inside and outside of the cell. Overall, the patterns of fold change for Ca^2+^-binding proteins suggest a significant increase in cytosolic Ca^2+ ^during blastema formation. Channel proteins in the plasma membrane that mediate extracellular Ca^2+ ^influx into the cytosol were upregulated on all dpa (CACNA1A, ATP11A) or at 7 days (CACNA2D3), while proteins that translocate Ca^2+ ^from the cytosol to the ER/SR (ATP2A3, SRL, ASPH), or buffer cytosolic Ca^2+ ^during muscle contraction (PVALB) were downregulated on all dpa. CAMK2D, a kinase that regulates transport of Ca^2+ ^into and out of cells, was downregulated at 4 and 7 dpa. Another kinase that is covalently linked to ion channels and regulates Ca^2+ ^influx is heart α-protein kinase (HAK), which was downregulated at 1 and 4 dpa, but strongly upregulated at 7 dpa. MYLC2PL, a mitochondrial Ca^2+ ^binding myosin light chain, was downregulated on all dpa. By contrast, CASQ1, which complexes to Ca^2+ ^for storage in the ER/SR and mitochondria, was upregulated on all dpa. Another protein upregulated at 4 and 7 dpa was the Ca^2+ ^binding mitochondrial solute carrier (SLC25A24), which shuttles metabolites, nucleotides and cofactors through the mitochondrial inner membrane.

An interesting group of calcium/phospholipid-binding proteins was the annexins. ANXA1, which is thought to reduce inflammation and promote fibrinolysis, was downregulated at 1 and 4 dpa. ANXA2 was upregulated at 1 and 4 dpa and ANXA4 and 6 were upregulated at 7 dpa. ANXA2 is an autocrine factor that promotes osteoclast formation and bone resorption, and ANXA4 and 6 promote exocytosis in epithelial cells.

### Transcription

A total of 58 proteins were associated with transcription. Changes in 14 chromatin-associated proteins were detected. In all, 10 of these were H1 and H2 histones, with four being upregulated on all dpa and two downregulated at all dpa. Another was upregulated at 1 dpa, returning to control level at 7 dpa. Of the remaining three histones, one was upregulated at 1 and 4 dpa before returning to control level at 7 dpa, another was downregulated at 1 and 4 dpa, but was upregulated at 7 dpa and the other showed no change at 1 dpa, then was downregulated at 4 and 7 dpa. Two proteins that regulate gene expression by covalent modification of histone proteins (MTA1) and nucleosome assembly (NAP1L1-A) were upregulated on all dpa and at 4 and 7 dpa, respectively. JMJD1B (Jumonji domain), a lysine-specific histone demethylase, was downregulated at all dpa (over sixfold at 7 dpa). Hairless (HR), a Jumonji domain-containing transcription factor that recruits histone acetylases to repress transcription, was upregulated at 1 and 4 dpa, and downregulated at 7 dpa.

Of the transcription-associated proteins, 21 were transcription factors. The majority of these were upregulated at all three or two of three dpa, particularly at 4 and 7 dpa. Of six factors expected to act in a general fashion, CBTF122, a subunit of the *Xenopus laevis *CCAAT box transcription factor, was the only one upregulated on all dpa. SND1 and TRIM29 were downregulated at 1 dpa but upregulated at 7 dpa while E4F1 and TAF4 were downregulated at 1 and 4 dpa and upregulated at 7 dpa. ATF1 was downregulated on all dpa. FUBP1, an ATP-dependent DNA helicase that stimulates *c-myc *expression in undifferentiated cells was upregulated at 7 dpa. MNT, an E-box (CANNTG) binding transcriptional repressor of *c-myc *was upregulated at 1 dpa, but downregulated at 4 and 7 dpa.

Six zinc finger transcription factors designated by number were noted, four of which were upregulated at all, or two of three dpa. Of the other two, ZNF777 was downregulated on all dpa, and ZNF559 was downregulated at 1 and 4 dpa, and then upregulated at 7 dpa. The Kruppel-like factor 6 is a ubiquitously expressed zinc finger tumor suppressor that was upregulated at 1 and 4 dpa, and then downregulated at 7 dpa. Several factors (AHCTF1, nuclear receptor subfamily 2, group C member 2 (NR2C2), nuclear factor of activated T-cells cytoplasmic 4 (NFATC4), sex determining region Y box 6 (SOX6), and LIN28 that were upregulated on all, or two of three dpa, induce transcription of specific sets of genes. For example, NR2C2 is a nuclear receptor for mineralocorticoids and glucocorticoids, NFATC4 plays a role in inducing cytokine gene expression in T cells, and SOX6 is required for neurogenic and skeletal differentiation. LIN28 is a transcription factor active in embryonic stem cells [48]. NEUROD2, a neuronal differentiation factor, was downregulated on all dpa.

In all, 23 of the transcriptional proteins were associated with mRNA processing. At 1 and 4 dpa, downregulation predominated over upregulation. By 7 dpa, however, the U/D ratio was 1.5. The majority of the processing proteins were heterogeneous nuclear ribonucleoproteins, small nuclear riboproteins, and splicing factors. One of these proteins, CWC15, was downregulated over threefold at 7 dpa. Two DEAD box helicases, which unwind RNA structure for accessibility by splicing enzymes, were detected. DEAD box polypeptide 10 (DDX10) was upregulated on all dpa, while DDX46 was upregulated at 1 dpa and downregulated at 4 and 7 dpa. MATR3 anchors mRNA to the nuclear matrix, and was upregulated on all dpa. RBM, a RNA-binding protein of unknown function, was upregulated at 4 and 7 dpa.

### Translation

Many of the 20 proteins involved in translation, particularly ribosome structural proteins, were upregulated. We detected 13 ribosome structural proteins, about evenly divided between the 60S and 40S subunits. Two of these, RPL7L1 and RPS20, were upregulated at all the time points. Factors for initiation (PABPC1), binding of mRNA to the ribosome (E1F4B), and translocation of nascent protein from the A site to the B site of the ribosome (EEF2) were downregulated or unchanged at 1 dpa, but were upregulated at 4 and 7 dpa. Another initiation factor, E1F4A1, was downregulated at 1 dpa, returned to control level at 4 dpa, and was upregulated at 7 dpa. The elongation factor EEF1A2 was upregulated on all dpa. TARSL2, which is involved in tRNA aminoacylation, was upregulated at 1 dpa, and downregulated at 4 and 7 dpa. Lastly, a translation termination factor, ETF1, was upregulated at 4 and 7 dpa.

### Cytoskeleton

About one-third of the cytoskeletal proteins were sarcomeric proteins of skeletal muscle, and these were heavily downregulated. Many, such as TNNT3A, TM7, myosin light chain 3 (MYL3) and MYL5, were downregulated at all the time points.

Of the 40 non-sarcomeric proteins, 25 had functions related to cell motility and maintenance of cell shape and structural integrity. The U/D ratio of these proteins strongly favored downregulation at 1 dpa, but the ratio shifted in favor of upregulation at 4 and 7 dpa. Proteins that were downregulated on all dpa were ACTN1 and 4, GOLGA1, PLS3, XAK-B, and cytokeratin type II. Proteins downregulated at 1 and 4 dpa were desmoplakin isoform II, KRT 12 and KRT5.5. NAV1 was upregulated at all dpa. Seven proteins, FLNB, **SYNE2**, TUBA, TUBA4B, KRT 19, ACTR2-A and TUBB2C, were downregulated or showed no change at 1 dpa, then were upregulated at 4 and 7 dpa. The remaining proteins MYO9A, MYH9, ACTG1, TUBB4, desmoplakin (DSP), XAK-C and EPPK1, showed a mixture of fold change patterns.

In all, 10 proteins are involved in intracellular movement. MYO1C and MYO5A were downregulated at 1 and 4 dpa, but upregulated at 7 dpa. DYNC1LI2 was upregulated at 1 and 4 dpa, but downregulated at 7 dpa, DNAH3 was downregulated at 4 and 7 dpa, and DYNLL1 was downregulated on all dpa. MYH1 was upregulated at 1, then downregulated at 4 and 7 dpa. MYO1E was upregulated at 1 dpa, downregulated at 4 dpa, and returned to control level at 7 dpa. Two proteins that move or anchor kinases to the cytoskeleton (PDLIM1, PALM2) were downregulated at 4 and 7 dpa. The major vault protein (MVP), which may act as a scaffold for kinases involved in signal transduction and may also play a role in nucleocytoplasmic transport, was downregulated at 1 and 4 dpa, returning to control level at 7 dpa.

There were five adhesion proteins. CDH5 (vascular endothelial cadherin), SCARF2, and ST3GAL5, a type II membrane protein that also maintains fibroblast morphology, were upregulated at all dpa, while CNTNAP4 and FHDC1were downregulated at all dpa.

Of the remaining five non-sarcomeric proteins, KPNA2, which is involved in the import of nuclear proteins, and MYOF, a Ca^2+^/phospholipid-binding protein that promotes rapid resealing of damaged endothelial cell membranes, were downregulated on 4 and 7 dpa. Sorbin (SORBS1), which plays a role in insulin-stimulated glucose transport, was downregulated on all dpa. By contrast, piccolo (PCLO), which organizes the cytoskeleton in synaptic zones, and PMFBP1, a general cytoskeletal organizing protein, were upregulated at all dpa.

### ECM

Components of collagen 1 and collagen 13 were upregulated at all or two of three dpa. Collagen 5 was upregulated at 1 and 4 dpa, and then downregulated at 7 dpa. Components of cartilage matrix (collagen 2) and basement membrane (collagen 4) were downregulated at all dpa, as was decorin, which interacts with collagen1 fibrils and may affect the rate of their formation. However, matrilin (MATN) 4, a major component of cartilage matrix, was upregulated at 1 and 4 dpa, then downregulated at 7 dpa. FBN1, a large glycoprotein that associates with elastin to provide force-bearing support in the ECM, was upregulated at 1 and 7 dpa, with no change at 4 dpa. MATN 2, a von Willebrand family member involved in matrix assembly, was upregulated at 1 and 4 dpa, then returned to control level at 7 dpa. FGB, FGG, and fibronectin 1 (FN1) form part of the provisional wound matrix (clot) and were upregulated at all dpa, whereas another provisional matrix protein, tenascin, was downregulated at 1 dpa, showed no change at 4 dpa, and was upregulated at 7 dpa. Periostin, an osteoblast specific factor, was downregulated at 1 and 4 dpa, but upregulated at 7 dpa. EHD4, an endosomal transport protein that promotes assembly and stabilization of collagen 6 filaments, showed no change at 1 dpa and was downregulated at 4 and 7 dpa. Tubulointerstitial nephritis antigen (TINAG), a basement membrane glycoprotein that mediates adhesion of proximal tubule epithelial cells via cell surface integrins, was downregulated on all dpa.

### Metabolism

Eight proteins directly or indirectly involved in oxidative phosphorylation were detected. ATP5B, COX-Va, ECHS1, GLUD1 and CS function in the citric acid cycle; most were downregulated at all or two of three dpa. The only mitochondrial metabolic protein that was upregulated at all dpa was SLC25A4, an adenine nucleotide translocator that catalyzes the exchange of adenosine di- and triphosphate (ADP and ATP) across the inner mitochondrial membrane, but a second translocator, SLC25A13, was downregulated at all dpa. Eight proteins involved in the glycolytic pathway were detected, most of which were downregulated at all or two of three dpa. Two proteins, PGM1 and PYGM, are involved in glycogen metabolism; both were downregulated at all dpa.

In all, 15 other metabolic proteins were detected. Most were downregulated at 1 and 4 dpa, with the U/D ratio rising to 1.00 at 7 dpa. Three exceptions were DAGLB, which catalyzes DAG to the endocannabinoid 2-arachidonoyl glycerol (2-AG), DHRS4, which is involved in retinoid metabolism, and PAPPA2 a matrix metalloproteinase that cleaves IGFBP-5. All were upregulated on all dpa.

### Cell protection

Seven proteins associated with the post amputation inflammatory response were antioxidants or antipathogens, proinflammatory enzymes, or detoxicants. The antioxidants PXDN and PRDX1 were upregulated on all dpa, while antioxidant TLR6 was upregulated at 1 and 4 dpa. OAS2 and GSTP1, which activate responses to pathogens, were upregulated at 4 and 7 dpa. The proinflammatory enzyme AOX1, by contrast, was downregulated at 1 and 4 dpa. CYP2F1, which plays a role in detoxification, was downregulated on all dpa.

A total of 13 apoptotic pathway-related proteins were detected. Six of these are involved in proapoptotic pathways, and all but one was downregulated on all or two of three dpa. The four downregulated proapoptotic proteins were MICB, a stress induced self-antigen that leads to cell lysis by T cells, VDAC1, a mitochondrial ion channel that promotes apoptosis when open, FASTKD5, which initiates caspase activity, and AK2, which is located in the mitochondrial intermembrane space. Exceptions were microtubule associated serine/threonine kinase 3 (MAST3), which was upregulated at 1 and 4 dpa, and ABTB1, which was upregulated at 4 and 7 dpa. ABTB1 mediates the phosphatase and tensin homolog (PTEN) growth-suppressive signaling pathway. Both negatively regulate the Akt cell survival pathway. Of the seven antiapoptotic proteins, three were downregulated at all or two of three dpa (AKT1S1, BIRC6, and PDCD6IP). Antiapoptotic proteins upregulated at two of three dpa were NEK11 (genotoxic stress reponse), tumor necrosis factor receptor-associated factor 1 (TRAF1; mediates antiapoptotic signals from TNF receptors), and PAIRBP1 (mediates the antiapoptotic action of progesterone in mammalian cells). Interleukin 7 receptor (IL7R), which blocks apoptosis during the differentiation and activation of T lymphocytes, was downregulated at 1 dpa and upregulated at 7 dpa.

A total of 15 proteins that promote or stabilize protein folding in the ER were detected. Four were isomerases. FKBP10 and P4HB were downregulated at all dpa and protein disulfide isomerase A3 (PDIA3) at 1 and 4 dpa. PPIA was upregulated at all dpa and PDIA6 was upregulated at 4 and 7 dpa. A total of 10 proteins were members of chaperone families that accelerate protein folding in the ER. Two of these were upregulated at all dpa (heat shock protein (HSP)B3, TOR1A), three were upregulated at 4 and 7 dpa (HSP90B1, HSP90AB2P, CCT2), one was upregulated at 1 dpa and downregulated at 4 and 7 dpa (HSP27), and two were downregulated at 1 and 4 dpa, but upregulated at 7 dpa (PCMT1, HSP90AA1). Two other chaperones (SSR1 and HSP90AA1) were downregulated at 1 dpa and upregulated at 7 dpa.

### Degradation

Misfolded or damaged proteins that cannot be salvaged are polyubiquinated in the ER, transferred to the Golgi, and then to a cytosolic complex of proteins called the 26S proteasome, where they are degraded [49]. In our samples, we detected seven proteins of the proteasome pathway. HACE1 (a ubiquitin protein ligase) was upregulated at all dpa, and ubiquitin specific protease 3 (USP3), was upregulated at 1 and 7 dpa. Ubiquitin-like modifier activating enzyme 1 (UBA1) was upregulated at 1 dpa and downregulated at 4 and 7 dpa. We detected four proteins that are part of the proteasome itself. Three of the four were upregulated only at 7 dpa (PSMB8, PSMD 2,7), whereas PSMC4 was downregulated at 1 and 4 dpa before returning to control level at 7 dpa.

Cell debris produced by histolysis, necrosis or apoptosis, is degraded by cytosolic proteases and lysosomal enzymes, and removed by exocytotic pathways. EXOC7, a component of the exocyst, a protein complex essential for docking exocytotic vesicles to the plasma membrane, was upregulated at all three dpa, suggesting the removal of degraded material by this pathway. Other degradative enzymes were TMPRSS9 (a serine protease) and membrane metalloendopeptidase (MME), both of which degrade small peptides. The former was downregulated at all dpa (by nearly sevenfold at 7 days), while the latter was upregulated at 1 and 4 dpa.

### Cell cycle

NME1, a kinase involved in the synthesis of nucleoside triphosphates other than ATP was upregulated at all dpa. MMCM3 (required for DNA replication) was downregulated at all dpa and FUS (a heterogeneous nuclear protein that promotes annealing of complementary DNA strands) was downregulated at 1 and 4 dpa, but upregulated at 7 dpa. Five cell cycle progression proteins were identified. WDR36 and MARK4 were downregulated on all dpa, whereas ULA1 was upregulated on all dpa. LOH11CR2A, a von Willebrand family member, acts as a tumor suppressor and a negative regulator of the cell cycle. It was downregulated at 1 and 4 dpa, returning to control level at 7 dpa. PPP1C, a protein phosphatase required for chromatin condensation and maintenance of histone H3 phosphorylation during mouse oocyte meiosis [50], showed no change at 1 dpa, and then was downregulated at 4 and 7 dpa.

Several proteins implicated in mitotic spindle formation were detected. CROCC, which contributes to centrosome cohesion before mitosis and NDEL1, which anchors microtubules to the centrosome during interphase and localizes to mitotic spindles during mitosis were upregulated on all dpa. However, XMAP215 and Ras-related nuclear protein (RAN), which regulate microtubule assembly during the cell cycle, were downregulated on all dpa and on 1 and 4 dpa, respectively. RAN has other functions as well, including translocation of RNA and proteins through the nuclear pore complex, DNA synthesis, and cell cycle progression. Titin (TTN), which in skeletal muscle serves as an adhesion template for the assembly of contractile machinery, and may play a role in chromosome condensation and segregation in non-muscle cells, was upregulated at 1 and 4 dpa. EVI5, a centrosomal oncoprotein implicated in the prevention of premature entry of cells into mitosis, and in the completion of cytokinesis, was upregulated at 1 dpa nearly to the level attained by NOS1, but unlike NOS1 its level remained exceptionally high at 4 and 7 dpa as well.

### Validation of proteomic methods

**Figure 4:**
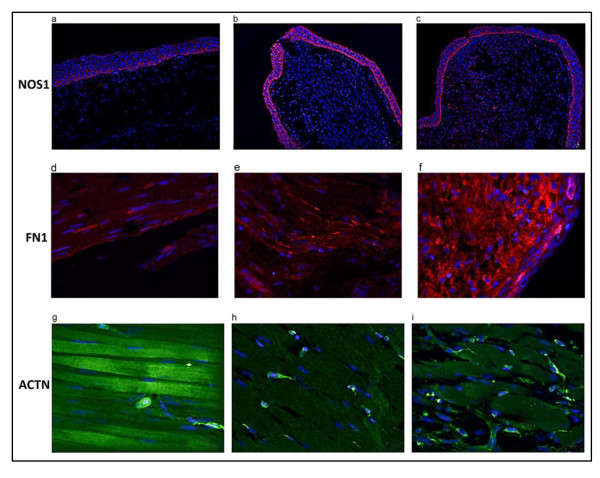
**Immunostained sections of axolotl hindlimbs**. Longitudinal sections of control **(a, d, g) **versus 1 day post amputation (dpa) **(b, e, h) **and 7 dpa **(c, f, i) **axolotl hindlimbs stained with primary antibodies to nitric oxide synthase 1 (NOS1) (a-c), fibronectin 1 (FN1) (d-f), α-actinin (ACTN) (g-i). Conjugated secondary antibodies were alexa-568 for fibronectin and NOS1, and alexa-488 for α-actinin. Nuclei were counterstained with 4',6-diamidino-2-phenylindole (DAPI). As expected from the proteomic data, fluorescence intensity of NOS1 showed a significant increase compared to control at 1 dpa, then decreased to a level slightly above control at 7 dpa. Fibronectin staining (red) at 1 and 7 dpa showed significant increases compared to controls, while α-actinin staining intensity (green) showed significant decreases.

Antibodies to axolotl proteins are not available. Most commercially available antibodies are directed against human and mouse antigens. We therefore tested antibodies to a variety of mammalian proteins that were upregulated in our study on control and regenerating limb tissue. Antibodies to three of these, NOS1, FN, and α-actinin, reacted strongly enough on longitudinal sections of axolotl limb tissue for direct validation by immunohistochemistry. We therefore tested the expression of these proteins at 1 and 7 dpa relative to control tissue (Figure 4). NOS1 and fibronectin were upregulated at 1 and 7 dpa, whereas α-actinin was downregulated. Table 3 shows the densitometric quantification of these proteins in immunostained sections. The fold changes determined by liquid chromatography/mass spectrometry/mass spectrometry (LC-MS/MS) were congruent with the densitometric measurements, indicating that quantitative LC-MS/MS data accurately reflect the levels of specific proteins. Indirect validation from the literature provides further support for this conclusion. For example, the upregulation of retinoids and chaperones observed in regenerating urodele limbs [51-53] matches a similar upregulation of DHRS4 and multiple chaperones in our study, as does the downregulation of citric acid cycle enzymes observed by Schmidt [54].

**Table 3:** Liquid chromatography/mass spectrometry/mass spectrometry (LC/MS/MS) versus densitometry measurements

Protein	Comparison	Fold change	
		
		LC-MS/MS	Densitometry
NOS1	0 dpa vs 1 dpa	4.93	2.0*
	
	0 dpa vs 7 dpa	1.16	1.2***

Fibronectin	0 dpa vs 1 dpa	1.39	1.51**
	
	0 dpa vs 7 dpa	1.46	3.17*

α-Actinin	0 dpa vs 1 dpa	-1.16	-0.62***
	
	0 dpa vs 7 dpa	-1.58	-0.83

Comparison of fold changes for NOS1, fibronectin and α-actinin with densitometry measurements on immunostained sections. Six images were collected for each immunostained section. Mean pixel intensities were calculated for each image by sampling 20 randomly-distributed regions of each image using the measurement package of the Axiovision software (Zeiss). Statistical comparisons were performed using ANOVA. A *P *value < 0.05 was considered statistically significant.

*P < 0.001; **P < 0.01; ***P < 0.05.

ANOVA = analysis of variance; dpa = days post amputation; NOS1 = nitric oxide synthase 1.

## Discussion

### Confidence in the methodology

We did not detect proteins such as Fgf-8, Hox a-d, sonic hedgehog, retinoic acid receptors, and matrix metalloproteinases whose transcripts are expressed during limb regeneration [10]. This could be due to an inability of current LC-MS/MS technology to confidently detect low abundance proteins, most of which [except for matrix metalloproteinases (MMPs)] are strongly expressed only at stages of regeneration beyond accumulation blastema (see [10] for review). However, the advantages of the method far outweigh this limitation. Firstly, proteomic analysis has the general advantage over genomic analysis of detecting the end products of gene activity, thus ignoring transcripts that may never be translated. Secondly, the LC-MS/MS-based label-free protein quantification technology used here has proven itself a powerful tool to resolve and identify thousands of proteins from complex biological samples [55,56]. It is a relative method that compares the expression level of the same protein under different conditions. The method is rapid and more sensitive than many other proteomic methods, and increases the protein dynamic range of threefold to fourfold compared to two-dimensional gel electrophoresis. During development of the method, chicken lysozyme was used as the quality assurance/quality control and the method has since been robustly tested on many different types of samples [56]. Automation allows it to be applied for large-scale proteomic analysis; thus it has become a tool of choice for biomarker discovery [57,58]. The inclusion of statistics in both experimental design and data analysis allows for the detection of small but significant changes not offered by other methods. We are thus confident in the qualitative and quantitative data produced in our study.

### Significance of results

#### Signaling and Ca^2+ ^binding and translocation

Myoinositol is a precursor to PIP2, which is cleaved into IP_3 _and DAG. IP_3 _stimulates a rise in cytosolic Ca^2+ ^that causes protein kinase C to translocate to the plasma membrane, where it is activated by DAG and regulates transcription [49]. Our data support the idea that an increase in myoinositol after amputation initiates signals that result in a major increase in cytosolic Ca^2+ ^by influx from extracellular sources or release from ER/SR stores. First, we found that ISYNA1, a key enzyme in the synthesis of myoinositol, is strongly upregulated. Second, we saw a general downregulation of proteins involved in Ca^2+ ^homeostasis, which would lead to a rise in cytosolic Ca^2+ ^that in turn would activate or suppress many different signaling pathways. One such downregulated protein was CAMK2D, which has also been implicated in the activation of enzymes such as NOS1 [59], regeneration of muscle fibers [60], and tissue repair [61]. Our data are consonant with the results of other studies indicating that inositol phosphates are generated from PIP2 within 30 s after amputation of the newt limb and that inhibiting their formation by beryllium prevents blastema formation [62,63]. They are also in harmony with studies showing that (1) intracellular Ca^2+ ^release in response to mitogenic signals is essential for mitosis in the newt limb blastema, [64-66], (2) protein kinase C (PKC) activity rises to a plateau at accumulation blastema to medium bud [67], (3) planarian regeneration is dependent on Ca^2+ ^[68], and (4) higher levels of several S100 family Ca^2+^-binding proteins are observed in the regenerating ear tissue of MRL/MpJ-Fas mice versus non-regenerating ear tissue of C57BL/6J mice, as determined by laser capture proteomics [69,70].

In addition to Ca^2+^, the translocation of other ions is essential for blastema formation in amputated amphibian limbs and tails. Ionic currents leave the newt limb immediately upon amputation, driven by Na^+ ^influx [71]. Proton efflux across the wound epidermis of the amputated *Xenopus *tadpole tail is driven by a vacuolar ATPase pump [72]. Vacuolar ATPases are expressed in the intracellular membranes (for example, lysosomes) of all eukaryotic cells [73], where they pump H^+ ^ions inward to maintain an acidic pH. The tadpole tail pump, however, is a plasma membrane v-ATPase [72]. Drug-induced inhibition of either Na^+ ^or H^+ ^movements results in failure of blastema formation [72,74]. A v-ATPase did not appear in our priority 1 or 2 sets of proteins, but was present in the priority 4 set. Furthermore, a protein subunit of a v-ATPase was detected in the stage 53 hind limb bud of *Xenopus *at 3 dpa, using methods identical to ours [75], and a gene encoding a v-ATPase was the most abundant clone in a suppressive subtraction cDNA library made from 4 dpa axolotl regenerating limb tissue [34]. Whether these are the same v-ATPases as the plasma v-ATPase of Adams *et al. *[72] is not known.

The annexins are phospholipid-binding signaling proteins that have been implicated in a variety of biological processes. Annexin 1 has been postulated to reduce inflammation in regenerating fish [76,77] appendages and in stage 53 regeneration-competent *Xenopus laevis *limb buds [75]. However, annexin 1 was upregulated only at 7 dpa in our samples. This expression pattern might reflect differences in the onset and/or persistence of the inflammatory phase of amputated axolotl limbs and *Xenopus *tadpole limb buds, differences in the immune systems of these species [78], or annexin 1 might have some other function in the accumulation blastema. Annexin 2 was upregulated at 1 and 4 dpa, and may be important for histolysis, since it has been shown to promote osteoclast formation and bone resorption [79]. This function correlates with the destruction of the periosteal bone shell by osteoclasts during blastema formation. Annexins 4 and 6 also were upregulated only at 7 dpa. These two proteins promote exocytosis in epithelial cells, consistent with the phagocytosis and elimination of debris by wound epithelial cells observed during early blastema formation [80].

NOS1 was the most strongly upregulated protein (4.93) at 1 dpa and was still upregulated relative to control at 4 and 7 dpa, although the fold change declined on each of these days. NOS1 catalyzes the synthesis of NO, which has a wide variety of signaling functions [81]. NO displays many properties of a neurotransmitter in the nervous system. It is produced by macrophages and neutrophils as a bactericidal agent, and has a role in activating proteases that are known to be important effectors of histolysis in regenerating limbs [82-85]. Immunostaining showed NOS1 to be expressed only in the epidermis over the period of blastema formation (Figure 4). This localization suggests that NO diffusing inward from the epidermis may be important to signaling pathways that regulate blastema formation. Grow *et al. *[31] found that the NOS1 gene was strongly upregulated in amputated stage 53 regeneration-competent *Xenopus *limb buds versus regeneration-deficient stage 57 limbs, suggesting that loss of NOS1 production is associated with loss of regenerative competence in *Xenopus *limb buds.

In addition to NOS1, other important signaling molecules and receptors, such as the nicotinic acid receptor, the insulin receptor, the ephrin receptor, tyrosine kinase 2, and GNB2L1, an anchor of PKC to the cytoskeleton, were upregulated on all or two of three dpa. Several Rab family GTPases and their activators and exchangers were differentially regulated. This family plays a critical role in regulating vesicle trafficking of proteins, including recycling of receptors, from one membrane compartment to another [86].

Five proteins involved in canonical or non-canonical Wnt signaling were detected. Wnt8 is considered a ligand for the canonical pathway, whereas other Wnt ligands seem to signal through the non-canonical pathway. These include Wnt3a and 4 in wound repair [87] and bone formation [88,89], and Wnt5a in *Xenopus *embryo convergent extension movements [90], and mouse embryo midgut elongation [91]. In our study, Wnt8 and APC were upregulated at 4 and 7 dpa. These are components of the canonical pathway that stabilizes β-catenin. Inversin switches the canonical pathway to the non-canonical pathway, by targeting the Disheveled protein for degradation by the proteasome or by the activation of the c-jun N-terminal kinase (JNK) pathway by DVL2 and axin [92]. Our results are consistent with the finding that Wnt genes for both pathways are expressed in the regenerating axolotl limb [93]. However, the fact that the DVL-binding protein CCDC88c, a negative regulator of the canonical pathway is upregulated on all dpa, and DIXDC1, a positive effector of the canonical pathway is downregulated on all dpa while inversin is upregulated over twofold by 7 dpa would suggest that regeneration in the axolotl limb might be promoted by the non-canonical Wnt pathway. By contrast, the canonical pathway (via Wnt8) was found to promote zebrafish fin regeneration whereas the non-canonical pathway inhibited it [94]. The canonical Wnt pathway has also been implicated in deer antler regeneration [95] and *Xenopus *tadpole tail regeneration [96]. Further studies will be required to understand the details of how Wnt signaling pathways regulate appendage regeneration in different species.

#### Transcription and translation

Previous studies of RNA and protein synthesis have shown that both increase during blastema formation, but do not reach maximum until differentiation of the new limb elements is initiated [36,37,39,41,97,98]. The U/D ratios for our data suggest that proteins involved in the transcriptional and translational machinery are generally upregulated, insuring that this machinery is available for whatever protein synthesis is required. However, the mRNA processing proteins appeared to be an exception, since their U/D ratio was quite low at 1 day, and did not rise above 1.0 until 7 dpa. This might mean that mRNA processing is a critical level of control for protein synthesis in general during blastema formation.

In addition, there were quantitative changes in chromatin proteins that suggest transcriptional changes by chromatin modification. The transcription factor hairless was upregulated at 1 and 4 dpa, possibly functioning to recruit histone deacetylases, and the histone lysine demethylase JMJD1B was downregulated sixfold at 7 dpa. Both of these patterns suggest transcriptional repression [99,100].

#### Cytoskeleton and ECM

The downregulation of sarcomeric proteins on all or two of three dpa, many over twofold, is consistent with cellularization of myofibers into mononucleate cells that undergo dedifferentiation [11,14,63,97]. The gradual rise in U/D ratio for motility, shape and structural integrity proteins at 4 and 7 dpa and the high U/D ratio for adhesion proteins such as SCARF2 and ST3GAL5, particularly at 1 and 4 dpa, is consistent with the migration of epidermal cells to close the wound, and the migration of dedifferentiating cells to accumulate under the wound epidermis. CDH5, a cadherin that mediates junctional adhesion of endothelial cells was upregulated at all dpa, perhaps reflecting the sealing of blood vessels and the initiation of new vessel formation.

The major vault protein (MVP) is the main component of vaults, large ribonucleoprotein particles that have been implicated in regulating cytoskeletal-associated kinase signaling [101-103]. The gene for this protein was upregulated in the established blastema of the regenerating zebrafish fin [104]. We found that the MVP was downregulated in amputated axolotl limbs at 1 and 4 dpa, but returned to control level at 7 dpa after an accumulation blastema was established. This fact, and the downregulation of another cytoskeleton-associated kinase, PDLIM1, may suggest less intracellular signaling by cytoskeletal-associated kinases during blastema formation.

With regard to ECM proteins, the upregulation of fibrinogen reflects formation of the fibrin clot. The upregulation of fibronectin and collagen 1, the downregulation of collagens 2 and 4, and the downregulation of EHD4, an endosomal trafficking regulatory protein [105] present in the matrix of differentiating cartilage and fibroblastic connective tissue during rat limb development [106], is consistent with other observations indicating that the differentiated tissue matrix is replaced by an ECM that is more similar to the limb bud matrix, and more favorable to the migration of dedifferentiated cells to form the blastema under the wound epidermis [107].

#### Metabolism and cell protection mechanisms

Amputation results in tremendous systemic and cellular stress. We found that DAGLB, which catalyzes the conversion of DAG to 2-AG, was highly upregulated on all dpa. 2-AG is required for axonal growth during development, and thus may play a role in nerve regeneration into the blastema, but it is also the most abundant endocannabinoid in adult tissues, suggesting its involvement in pain control during blastema formation. Our data are thus consistent with previous studies indicating that endorphins are upregulated after newt limb amputation [108,109]. The evolution of such painkilling mechanisms in urodele salamanders can be interpreted in terms of an adaptive response to the frequent cannibalization of limbs in the wild that occurs under conditions of crowding or inadequate food.

A major result of cell stress is apoptosis. Our data suggest that stress caused by amputation activates mechanisms to protect cells from apoptosis in regenerating axolotl limbs. The blastema forms under largely avascular, and thus hypoxic conditions [14,110] that could lead to apoptosis. Mammalian cells deal with hypoxia by upregulating hypoxia induced factor 1A (HIF1a), which regulates numerous downstream genes, including the PI3 kinase-dependent cell survival gene *Akt *and glycolytic enzymes to maintain ATP production [111-113]. Mammalian cells that fail to maintain ATP synthesis under hypoxic conditions are subject to apoptosis [114].

Naviaux *et al. *[115] compared metabolism in fibroblasts of the MRL/lpj mouse, which regenerates ear and heart tissue [7] versus the non-regenerating B6 mouse. They found that MRL fibroblasts exhibited the Warburg effect [116], a major feature of embryonic cell metabolism shared by cancer cells and cells involved in adult wound healing [117,118]. The Warburg effect is the increased reliance on glycolytic metabolism while maintaining normal O_2 _consumption. In spite of reduced energy production by oxidative phosphorylation, the number of mitochondria was higher in MRL than B6 cells, suggesting an under utilized functional reserve capacity [115]. Gorsic *et al. *[34] detected significant upregulation of the genes for cytochromes *b *and *c *and intense antibody staining to these cytochromes in the epidermis and underlying tissue of 4 dpa regenerating axolotl limbs, suggesting a similarity between axolotl and MRL cells in terms of mitochondrial enhancement.

Our data indicated that citric acid cycle and electron transport enzymes are downregulated on all or two of three dpa, consistent with previous studies showing a marked decrease in O_2 _usage during blastema formation in regenerating urodele limbs [119] and the histochemical absence of citric acid cycle enzymes [54,120]. Schmidt [54] proposed that the early blastema relies on anaerobic glycolysis or alternate pathways such as the pentose phosphate shunt and lipid metabolism to maintain ATP production. However, in our samples most of the glycolytic enzymes detected were downregulated throughout blastema formation. NO inhibits glycolysis and electron transport in skeletal muscle [121]. Thus the upregulation of NOS1, particularly at 1 dpa, might play a significant role in metabolic depression. A decrease in muscle metabolism during myofiber fragmentation and cellularization would account for much of this depression. Enough ATP production would remain, however, to synthesize the proteins necessary for epidermal wound healing, histolysis, and dedifferentiation. Lastly, one of the more strongly upregulated proteins on all dpa was DHRS4, which is involved in the reversible reduction of all-trans and 9-cis retinal. This upregulation is consistent with the important roles retinoids play, not only in metabolism, but also in the patterning of the blastema [51,122]. The role of specific metabolic changes in blastema formation merits revisitation.

Our histological observations indicated little cell apoptosis on 4 and 7 dpa, consistent with the results of terminal deoxynucleotidyl transferase dUTP nick end labeling (TUNEL) assays [123,124]. We propose that apoptosis is minimized by reducing metabolism and engaging protective mechanisms that include the upregulation of antimicrobial and antioxidant proteins, the differential regulation of proapoptotic and antiapoptotic proteins, and the unfolded protein response (UPR). The UPR is a response to cell stress caused by the accumulation of unfolded proteins within the ER/SR due to loss of Ca^2+ ^homeostasis, inadequate disulfide bond formation of nascent proteins by isomerases, or deficient protein glycosylation [49,125,126]. The UPR counters this stress in several ways: reducing the amount of protein translocated into the lumen, increasing protein degradation by proteasomes and exocytotic mechanisms, and increasing the capacity to accelerate protein folding in the ER by upregulating isomerases and chaperones. Failure to refold misfolded proteins or remove them from the ER results in apoptosis.

Our evidence for this idea is as follows. Firstly, antimicrobial and antioxidant proteins were consistently upregulated, and proinflammatory enzymes downregulated on most dpa. Secondly, four of five proapototic proteins were downregulated on all or two of three dpa. Conversely, four of seven antiapoptotic proteins were upregulated in the same pattern, although the AKTS1 protein, a substrate for the Akt survival enzyme, was downregulated on all dpa. Thirdly, the upregulation of two isomerases and several chaperones on all or two of three dpa suggests that the regenerating limb mounts an UPR. The upregulation of chaperone genes has been reported in other studies of regenerating newt and axolotl limbs [35,52,53], *Xenopus *stage 52 hindlimbs [33], and zebrafish fins [76,104]. Interestingly, in *Xenopus *limb buds rendered regeneration deficient by heat shock induced expression of transgenic *noggin*, chaperone gene expression is not maintained as it is in wild-type buds [33]. Gorsic *et al. *[34] reported the upregulation of two genes associated with combating cell stress in regenerating axolotl limbs at 4 dpa. These were *Sara1b*, a Ras-related gene whose product is involved in protein transport from the ER to the Golgi, and *Hmox-1*, which increases tolerance to hypoxia and protects against apoptosis [127]. This enzyme is also upregulated during liver regeneration [128].

### Dedifferentiation

Dedifferentiation occurs in conjunction with the liberation of cells from their tissue matrix by protease-induced histolysis. Dedifferentiated cells express a number of genes associated with the dedifferentiated state, such as *msx1 *[129], *Nrad *[130], *rfrng *and *notch *[131]. Nuclear transplantation studies [132] and ectopic grafting experiments [133] have shown that blastema cells are not reprogrammed to pluripotency. However, three of the four transcription factor genes (*Klf4, Sox2, c-myc*) used to reprogram mammalian adult somatic cells to pluripotency [48,134] are upregulated during blastema formation in regenerating newt limbs, and also during lens regeneration [135]. Beyond this, little is known about the molecular mechanism of dedifferentiation in the regenerating urodele limb. Interestingly, we found that LIN28, a fourth transcription factor used to reprogram mammalian somatic cells to pluripotency [48], was upregulated on all dpa. Thus it is possible that LIN28 might play a role in the transcriptional regulation of nuclear reprogramming during limb cell dedifferentiation. The molecular characterization of blastema cell surface antigens and study of the regulation of dedifferentiation by transcription factors, microRNAs, polycomb proteins and chromatin-modifying enzymes will be crucial for understanding the mechanism of dedifferentiation in regenerating amphibian limbs.

In a recent meeting review, Tanaka and Galliot [136] described data presented by Andras Simon indicating that activation of apoptotic pathways in cultured newt myotubes resulted in their cellularization, suggesting that these pathways might play a role in dedifferentiation. Our data suggest both positive and negative regulation of apoptotic pathway proteins. We suggest that some apoptotic pathways involved in eliminating internal structure (dedifferentiation) are selectively activated, while others that would destroy nuclei and plasma membranes are selectively downregulated. Evidence from other systems is consistent with this idea. Firstly, treatment of cultured insulin-producing INS-1E cells with the reversible ER stress inducer cyclopiazonic acid (CPA) upregulated genes related to ER stress while simultaneously downregulating genes related to differentiated β-cell functions [137]. Secondly, NO signaling inhibits apoptosis and induces dedifferentiation of chondrocytes *in vitro *via p38 kinase and calveolin 1 [138,139]. The UPR is induced in mice transgenic for a mutation that leads to accumulation of misfolded collagen 10 α1 (X) chains in the hypertrophic chondrocytes of developing endochondral bones [140]. However, instead of undergoing apoptosis, the chondrocytes undergo dedifferentiation, with re-expression of genes characteristic of a prehypertrophic state and re-entry into the cell cycle. Thirdly, paraquat treatment causes oxidative stress that induces the apoptosis of retinal photoreceptors and amacrine neurons *in vitro*, but promotes dedifferentiation of Muller glial cells, which have been proposed as a source of retinal stem cells [141]. Furthermore, coculturing retinal neurons with glial cells prevented paraquat-induced apoptosis. These results suggest that oxidative stress may activate Muller glia to both protect and replenish retinal neurons. Fourthly, newt and chick embryo retinal pigmented epithelial (RPE) cells can dedifferentiate and then become neurons or lens cells [142-144]. Dedifferentiation of chick embryo RPE cells is dependent on a rise in intracellular Ca^2+ ^[145] and neuronal Na^+ ^and Ca^2+ ^channels have been detected in cultured newt RPE cells [146]. Lastly, cell stress induces dedifferentiation and an epithelial to mesenchymal (EMT)-like phenotype in cultured PC C13 thyroid cells [147]. The relationship between apoptosis and dedifferentiation is thus another potentially exciting avenue of regeneration research.

### Cell cycle proteins and blastema formation

**Figure 5:**
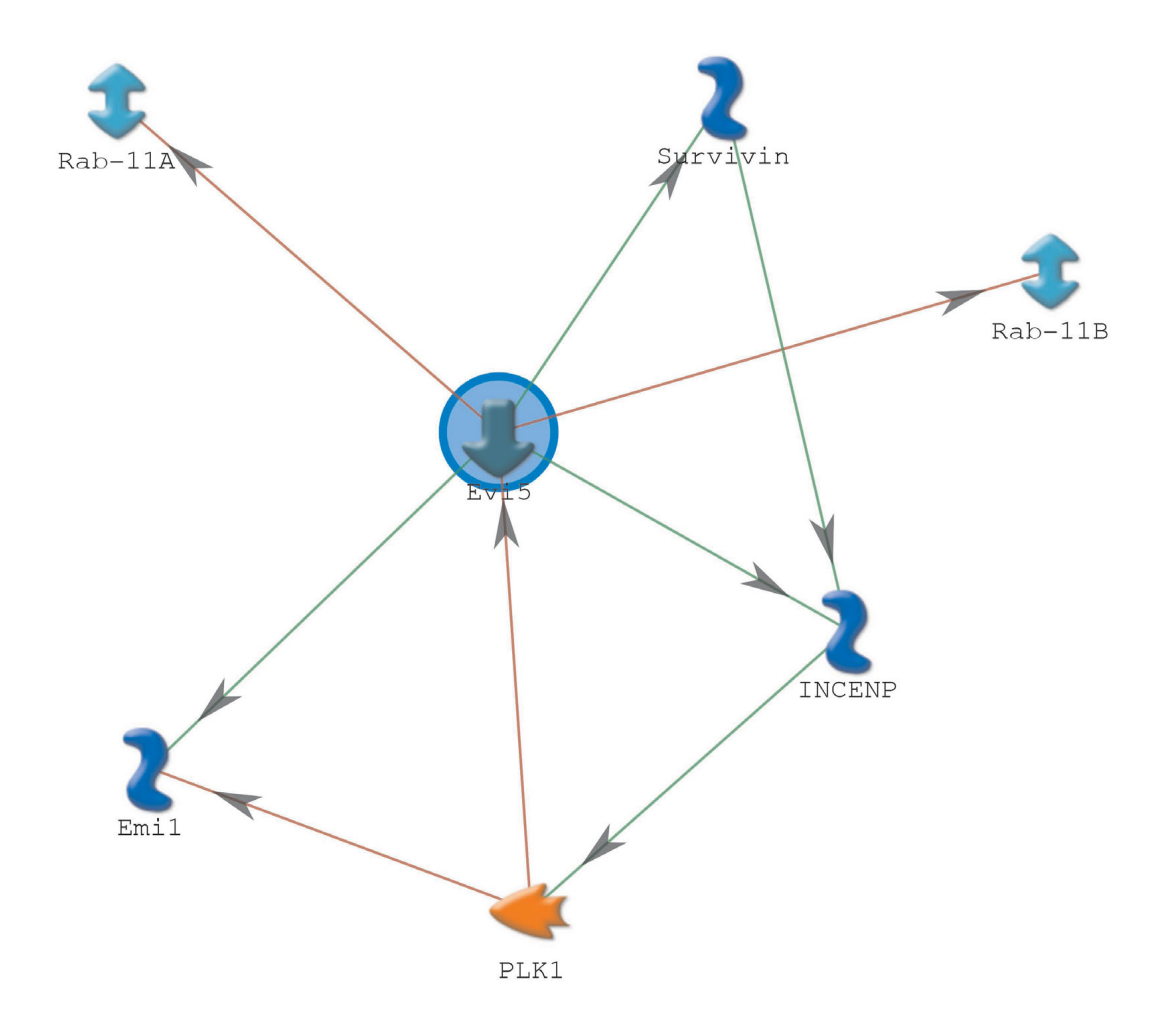
**Ecotropic viral integrative factor 5 (EVI5) network**. Network of direct interactions of six proteins with EVI5. Green = positive regulation; orange = negative regulation. The 110-kDa form of EVI5 stabilizes Emi1 to prevent premature entry into mitosis. At the same time, EVI5 inhibits the vesicle trafficking function of Rab 11a and b. Polo-like kinase 1 (PLK1) is then activated by inner centromere protein (INCENP) to degrade both EVI5 and Emi1, allowing progression into mitosis (M). During M, 90-kDA and 20-kDa forms of EVI5 interact with the chromosomal passenger complex (CPC) proteins aurora B kinase, INCENP, and survivin, where EVI5 is necessary for cytokinesis.

In all, 14 proteins associated with the cell cycle were detected. Of these, EVI5, the ecotropic viral integration site 5, was of interest because it was the most strongly upregulated protein over all dpa. EVI5 is a centrosomal oncoprotein that has several forms that interact directly with several other proteins in the cell cycle [148] (Figure 5). The 110-kDa form of EVI5 accumulates in the nucleus during early G_1. _It prevents cells from prematurely entering mitosis by stabilizing Emi1, a protein that accumulates in late G_1 _and inhibits cyclin A degradation by the anaphase-promoting complex/cyclosome (APC/C), allowing the cells to traverse S [149]. Emi1 and EVI5 are then targeted for ubiquitin-driven degradation after being phosphorylated by Polo-like kinase 1 (PLK1), allowing the cell to enter mitosis. The 110-kDa form of EVI5 may be degraded into 90-kDa and 20-kDa forms that at anaphase become associated with the chromosomal passenger complex (CPC) consisting of aurora B kinase, inner centromere proteins (INCENP), and survivin [148]. At late telophase and cytokinesis, EVI5 dissociates from the CPC and localizes in the region between the two daughter cells. Knockdown of EVI5 inhibits cytokinesis and results in the formation of binucleate cells [148]. EVI5 also renders the vesicle trafficking protein Rab 11 inactive, which would help restrain cells from entering mitosis by inhibiting the vesicular recycling of growth factor receptors that would otherwise promote the transduction of mitotic signals [150,151].

An interesting role for EVI5 in blastema formation can be postulated based on its functions in the mammalian cell cycle. Histological [17-19,152,153], electron microscopic [97] and genetic marking [154] studies indicate that cells located within the histolytic region of amputated urodele limbs begin to dedifferentiate within 2 dpa. Chalkley [17,18] showed that cell number during histolysis in this region is highest just proximal to the amputation plane. However, the mitotic index during histolysis is very low (0.1% to 0.5%) [17,20,21]. Coincident with the appearance of the blastema, the high point in cell number moves distal to the amputation plane, indicating that the blastema forms primarily by the distal migration and accumulation of dedifferentiated cells under the wound epidermis [17]. The mitotic index of blastema cells rises significantly only after the accumulation blastema has formed.

The cycle time of axolotl blastema cells at stages later than accumulation blastema is 40 h, with 39 h (approximately 1.5 days) spent in G_1_/S/G_2 _[22]. The high level of EVI5 during blastema formation suggests that it extends (by stabilizing Emi1) the premitotic portion of the cell cycle beyond 39 h for whatever period of time is required to form an accumulation blastema (in our case, 7 days). EVI5 would then be cleaved, the cells would traverse M and continue to cycle on the neural and epidermal-dependent 40 h time scale. This hypothesis makes two predictions. Firstly, only the 110-kDa form of EVI5 would be detected in blastema cells during formation of the accumulation blastema, but that the 90-kDa and 20-kDa forms would also be detected, in association with CPC proteins, as normal cycling began. Secondly, denervation or blocking contact of blastema cells with the wound epidermis, either of which inhibits blastema cell mitosis at any stage of regeneration [23], would cause cell cycle arrest, most likely in S or G_2 _phases, due to maintenance of high levels of EVI5. The role of neural and epidermal factors in mitosis, such as nAG [24] and Fgf-8 [155] would then be to signal for the cleavage of EVI5.

Interestingly, the CPC protein aurora B kinase appears to dissociate HP1 proteins from methylated histone H3 at the onset of mitosis [156-158], and is required for chromatin remodeling during postmitotic differentiation of mesenchymal stem cells and B cells [159]. Aurora B kinase maintains C2C12 cells in a differentiated state by phosphorylating serine 10 of histone 3 [160]. The small synthetic molecules reversine and hesparadin inhibit this phosphorylation, silencing muscle regulatory factor genes and inducing the inhibitor of differentiation gene, an induction that involves decreasing the methylation of histone H3 lysine 9 and increasing overall H3 acetylation. Treated C2C12 cells are then able to differentiate into adipocytes and osteoblasts [160].

The role of EVI5 and CPC proteins in the cell cycle and their relation to chromatin structure, dedifferentiation, and differentiation during regeneration will be an interesting avenue to explore.

## Conclusion

**Figure 6:**
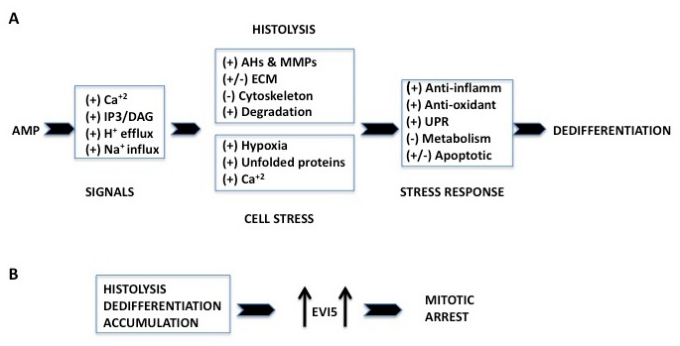
**Summary diagram of regeneration processes**. **(a) **Amputation generates signals that result in histolysis and liberation of cells from their tissue matrix. At the same time, these cells are under hypoxic and endoplasmic reticulum (ER) stress, and use a variety of mechanisms to counter this stress and prevent apoptosis, including upregulation of antiapoptotic pathways that protect cell membranes and nuclei. Some proapoptotic pathways are upregulated but are co-opted to remodel or eliminate internal cell structure. Along with changes in transcription factors, chromatin modifying enzymes, microRNAs and polycomb proteins, these mechanisms lead to dedifferentiation. **(b) **Throughout histolysis, dedifferentiation and accumulation of blastema cells under the wound epidermis, ecotropic viral integrative factor 5 (EVI5) is highly upregulated, preventing blastema cells from undergoing mitosis until after the accumulation blastema has formed.

Figure 6 depicts a model of regeneration based on integrating our findings with those of others. We recognize that many of the proteins detected in this analysis have multiple functions, and that their roles can therefore be subject to more than one interpretation. Thus we are pursuing a systems biology approach to use the high-abundance proteins revealed in our analyses as bait to retrieve associated low-abundance proteins from the literature and construct all possible protein networks and pathways involved in successful limb regeneration. Which of these pathways are correct, and their precise roles, can then be tested by loss and gain of function experiments.

We have also analyzed the fold changes of proteins during pseudoblastema formation in the amputated *Xenopus *froglet hindlimb, which regenerates only a muscle-less spike of cartilage [161]. The findings of this analysis will be compared to those reported here on the axolotl in order to gain insights into why the anuran limb bud loses the capacity for regeneration as it differentiates. This information will be useful in devising chemical induction strategies to reprogram mammalian somatic cells or activate resident stem cells directly at the site of injury to regenerate damaged tissues and appendages [28].

## Methods

### Animal surgery and tissue collection

All surgical procedures and animal care were carried out according to the Association for Assessment and Accreditation of Laboratory Animal Care (AALAC) standards followed at Indiana University-Purdue University Indianapolis (IUPUI), Purdue, IN, USA. *Ambystoma mexicanum *(axolotls) were obtained either by in-house breeding, or from the axolotl colony, University of Kentucky, Lexington, KY, USA. Animals 8 to 11 cm in length were anaesthetized in 0.02% to 0.05% MS-222 (Argent Chemical Laboratories, Redmond, WA, USA) and hind limbs were amputated bilaterally at mid-tibia/fibula. The tissue removed distal to the amputation site (what would be regenerated) served as the 0-day control. The regenerating tissue, along with a sliver (approximately 1 mm) of stump tissue, was collected at 1 day (epidermal wound healing), 4 days (histolysis and dedifferentiation) and 7 days (accumulation blastema) post amputation (dpa). The tissues were rinsed in sterile phosphate buffered saline (PBS) and flash frozen for proteomic analysis, which was conducted by Monarch Life Sciences (Indianapolis, IN, USA).

### Histology, immunostaining and image analysis

For histology, control and regenerating limb tissues at 1, 4, and 7 dpa were fixed in Bouin's solution for 48 h. Fixed tissues were then washed in 50% alcohol to remove the picric acid and stored in 70% alcohol. The tissues were dehydrated in a graded series of alcohols to 100%, followed by two changes of xylene for 45 min to 1 h each, after which they were infiltrated overnight with Pararaplast (Fisher Healthcare, A Fisher Scientific Company, Houston, TX, USA). The tissues were then embedded in fresh Paraplast and sectioned at 10 μm. Sections were stained with Weigert's iron hematoxylin and light green SF yellow and photographed at 10 × magnification on a Nikon Eclipse E800 microscope (Nikon Instruments Inc, Melvlle, NY, USA).

For immunostaining, control and regenerating limb tissues were collected at 1 and 7 dpa and fixed overnight in 2% paraformaldehyde in 0.8 × PBS. The samples were then rinsed with 1.0 × PBS and decalcified for 30 min using immunoclear decalcifying agent (Calci-Clear Rapid, National Diagnostics, Atlanta, GA, USA). After decalcification, the samples were cryoprotected by sequential overnight incubation in 10%, 20% and 30% sucrose in 1 × PBS, then embedded in a 50:50 mixture of 30% sucrose and Neg 50 frozen section medium (Thermo-Fisher Scientific, Waltham, MA, USA). Sections were cut at 10 μm on a Leica CM1900 cryostat (Leica, Wetzlar, Germany) and incubated in 1 × PBS to remove excess embedding medium, then blocked for 30 min in a solution of 0.01% Tween-20 and 5% milk in tris(hydroxymethyl)aminomethane (Tris)-buffered saline. Sections were then incubated over night with polyclonal anti-rabbit NOS1 (Biomol International LP, Plymouth Meeting, PA, USA) at 1:70 dilution, polyclonal anti-human fibronectin (Sigma, St Louis, MO, USA) at 1:400 dilution or monoclonal anti-α-actinin (Sigma) at 1:200 dilution, washed with blocking solution, incubated in the appropriate secondary antibody (goat anti-mouse AF488 or goat anti-rabbit AF568, Invitrogen, Carlsbad, CA, USA) for 40 min, washed with 1 × PBS and mounted with Vectashield mounting medium containing 4',6-diamidino-2-phenylindole (DAPI; Vector Laboratories, Burlingame, CA, USA).

Immunostained sections were observed using the 20 × objective lens on a Zeiss Axiovert 200 M microscope (Carl Zeiss Microimaging, Thornwood, NY, USA) equipped with an apotome for optical sectioning, and images were captured with an Axiocam MRM high-resolution camera. Sections were obtained from two hindlimbs of three animals for each time point. Six images were collected for each section, from regions located at the tip of the amputated limb to just proximal to the plane of amputation for 1 and 7 dpa samples and across the putative amputation plane in control sections. Mean pixel intensities were calculated for each image by sampling 20 randomly distributed regions of each image using the measurement package of the Axiovision software. Regions of sections containing bone were omitted from analysis, as some bone tissue displayed autofluorescence. Statistical comparisons were performed using analysis of variance (ANOVA). A *P *value < 0.05 was considered statistically significant.

### Proteomic analysis

#### Sample preparation

A total of five pools of tissue each from control, 1 dpa, 4 dpa and 7 dpa limbs were collected. Each pool contained six tissues (from two hindlimbs of three animals). The samples were processed as described previously [57]. Briefly, flash-frozen tissues were homogenized in lysis buffer containing 8 M urea and 10 mM dithiothreitol (DTT). The resulting cell lysates were denatured by urea, reduced by triethylphosphine, alkylated by iododethanol and digested by trypsin. The BCA Protein Assay (Bio-Rad, Hercules, CA, USA) was used to determine the peptide concentration in each pool.

#### LC-MS/MS analysis

Tryptic digested peptides were analyzed as previously described [57]. Samples were run on a Surveyor high performance liquid chromatography (HPLC) system (Thermo-Fisher Scientific) with a zorbax 300SB-C18 reverse column (1 mm × 5 cm). Each peptide pool (20 μg) was injected twice onto the column in a random order. All injections were performed using the identical equipment configuration. Peptides were eluted with a gradient from 5% to 45% acetonitrile developed over 120 min at a flow rate of 50 μl/min, and effluent was electrosprayed into the LTQ mass spectrometer (Thermo-Fisher Scientific). Data were collected in the 'TriplePlay' mode (MS scan, zoom scan, and MS/MS scan). The resulting data were filtered (to increase the signal to noise ratio) and analyzed by a proprietary algorithm developed by Higgs *et al. *[162].

#### Protein identification

Using SEQUEST (Thermo Fisher Scentific, Waltham, MA, USA) and X! Tandem (an open source algorithm provided by The Global Proteome Machine Organization http://www.thegpm.org database search algorithms, database searches against non-redundant (NR) National Center for Biotechnology Information (NCBI) or International Protein Index (IPI) databases were performed for peptide sequence identification. A confidence score was assigned to each peptide by q value (false discovery rate) [162]. The score was based on a random forest recursive partition supervised learning algorithm. The percentage ID confidence score was calibrated so that approximately X% of the peptides with percentage ID confidence >X% were correctly identified [162].

Proteins were classified according to identification quality (priority). This priority system is based on the quality of the amino acid sequence identification (peptide ID confidence) and whether one or more unique peptide sequences were identified (multiple sequences). The peptide id confidence assigned a protein into 'high' or 'moderate' categories based on the peptide with the highest peptide ID confidence (the best peptide). Proteins with 'best peptide' having a confidence between 90% to 100% were assigned to the 'high' category while proteins with best peptide having a confidence between 75% to 89% were assigned to the 'moderate' category. All peptides with confidence less than 75% were discarded. To increase the confidence in protein identification, the proteins were further classified based on the number of distinct amino acid sequences identified. A protein was classified as 'yes' if it had at least two distinct amino acid sequences with the required ID confidence; otherwise it was classified as 'no'. Thus, the proteins with 'high' peptide ID confidence and with more than one identified peptide sequence were termed priority 1. Proteins with 'high' peptide confidence but with only one identified peptide sequence were termed priority 2. Priority 3 and 4 proteins were those with 'moderate' peptide confidence with more than one and only one peptide sequence identified, respectively. Thus, priority 1 proteins had the highest likelihood of correct identification and priority 4 proteins the lowest likelihood of correct identification.

#### Protein quantification and statistical analysis

Protein quantification was carried out using non-gel based and label-free proprietary protein quantification technology described previously [57,162]. All measurements on experimental samples reflect up or downregulation, or no change, relative to control samples. Every peptide quantified had an intensity measurement for every sample. This measurement is a relative quantity giving the area under the curve (AUC) from the extracted ion chromatogram (XIC) after background noise removal. The AUC was measured at the same retention time window (1 min) for each sample after the sample chromatograms had been aligned [162]. The intensities were then transformed to the log base 2 scale (commonly used for genomic data), which served several purposes. First, relative changes in protein expression are best described by simple ratios. However ratios are difficult to model statistically, so log transformation converts ratios to fold differences. Second, the transformed data better approximate a normal distribution on a log scale [163], which is important because normality is an assumption of the ANOVA models used to analyze this data. Third, log base 2 is easy to understand because a twofold change (or doubling, or 100% increase) yielding an expression ratio of 2 is transformed to 1 (that is, a twofold change is a unit change on the log base 2 scale). After log transformation the data were then quantile normalized [164]. This normalization removed trends introduced by sample handling, sample preparation, HPLC, mass spectrometry, and possible total protein differences.

If multiple peptides had the same protein identification, their quantile normalized log base 2 intensities were weight averaged proportionally to their relative peptide ID confidences. Then, the log base 2 protein intensities were fitted by a separate ANOVA statistical model for each protein. Finally, the inverse log base 2 of each sample mean was calculated to determine the fold change (FC) between samples. The maximum observed absolute FC was also given for each priority level. FC was computed as mean regeneration group/mean control group. A FC of 1 means no change.

The number of proteins with significant changes for each priority was calculated. The threshold for significance was set to control the false discovery rate (FDR) for each two-group comparison at 5% [165]. The FDR was estimated by the q value, as stated previously. Thus protein fold changes with a q value less than or equal to 0.05 were declared to be significant, leaving 5% of the determined changes assumed to be false positives.

We calculated the median percentage coefficient of variance (%CV) for each priority group. Percentage CV values were derived from the standard deviation divided by the mean on a percentage scale. The percentage CV was calculated for replicate variation (technical variation) and the combined replicate plus sample variation.

In constructing biological process categories, only proteins having peptide confidence levels of 90% and above and with FDR < 0.05 were included. Many proteins were identified either by the same sequences or different sequences in priority 1 or 2 or both. To avoid redundancy, the fold changes of priority 1 were used if a protein was present in both the priorities, and average fold change was calculated if it belonged to same priority. If a protein had conflicting expression patterns (upregulated in one case, but downregulated in the other) then it was not considered.

### Bioinformatic analysis

Proteins not recognized by the algorithm were manually curated. NCBI blastp (basic local alignment search tool for proteins) [166] was used to match the sequences of hypothetical/novel/unknown/unnamed/Locus (LOC)/NIH Mammalian Gene Collection (MGC) proteins against the 'vertebrata' category in blast (taxid: 7742) to identify their closest neighbors. Only the proteins having 90% peptide ID confidence and above and with FDR < 0.05 were chosen. Accession numbers, gene names and names of the proteins were obtained from Uniprot [167] or NCBI [168] using the protein IDs obtained in the raw data. GeneCards [169] and Uniprot were used to determine their biological processes. The Human Protein Reference Database (HPRD) [170] was used to determine molecular function and primary cellular localization. The EVI5 network was generated using MetaCore analytical suite version 5.3 (GeneGo, St Joseph, MI, USA). Cluster 3.0 [171] and Java Treeview software [172] available from Stanford University were used to generate the global intensity expression map.

All non-redundant peptides having a peptide ID confidence of 90% and above were compared against expressed sequence tag (EST) contigs from the *Ambystoma *ESTdb (SR Voss) using tBLASTn.

## Authors' contributions

Project conception and experimental design: DLS, FS, NR, DJ. Staging and tissue harvest: DLS, NR, FS. Development and application of LC-MS/MS protein analysis: MW. Protein biological process/function/location search: DJ, NR, BL, DJM, HLDN, BS. Bioinformatics tools and analysis: DJ, MP. Data analysis and interpretation: NR, DJ, DJM, BL, DLS. Axolotl EST sequence matching: SRV. Histology, immunostaining, densitometry: NR, DJM. Manuscript writing: DLS, NR, DJ. Manuscript editing and critique: FS, BL, MWK, JAC.

## Supplementary Material

Additional file 1 **Statistically significant priority 1 and 2 proteins**. Each peptide is listed with its priority number, accession number, gene name, protein name, peptide sequence and maximum fold change (MFC). Proteins identified by axolotl ESTdb are in bold. Click here for file

Additional file 2 **Fold changes of 309 priority 1 and 2 proteins at 1, 4 and 7 days post amputation (dpa) relative to control for all categories of biological process**. Minus indicates negative fold change; otherwise the fold change is positive. NC = no change. Click here for file
